# Correction: The primary cilium dampens proliferative signaling and represses a G2/M transcriptional network in quiescent myoblasts

**DOI:** 10.1186/s12860-024-00513-9

**Published:** 2024-06-07

**Authors:** Nisha Venugopal, Ananga Ghosh, Hardik Gala, Ajoy Aloysius, Neha Vyas, Jyotsna Dhawan

**Affiliations:** 1 CSIR-Centre for Cellular and Molecular Biology, Uppal Road, Hyderabad , 500 007 India; 2 Academy of Scientific and Innovative Research, Ghaziabad, 201002 India; 3 National Centre for Biological Sciences, Bengaluru, 560065 India; 4 Present address: St. John?s Research Institute, Bengaluru, 560034 India; 5 Institute for Stem Cell Science and Regenerative Medicine, Bengaluru, 560065 India

**Correction: BMC Mol and Cell Biol 21, 25 (2020)**.

10.1186/s12860-020-00266-1.

Following publication of the original article [1], the authors reported an error in the affiliations of author Jyotsna Dhawan. Jyotsna Dhawan is also affiliated to institution 2, Academy of Scientific and Innovative Research, Ghaziabad 201,002, India.
