# Supplementary figures and images for: The primary cilium dampens proliferative signaling and represses a G2/M transcriptional network in quiescent myoblasts
Source: BMC Mol Cell Biol. 2020 Apr 15;21:25. doi: 10.1186/s12860-020-00266-1 (PMC7161131; doi:10.1186/s12860-020-00266-1)

A

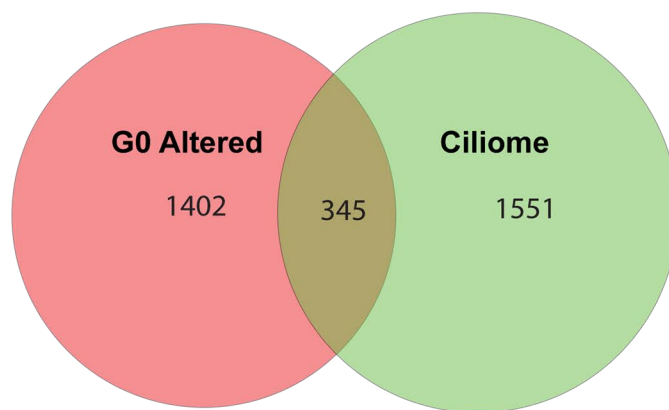

B

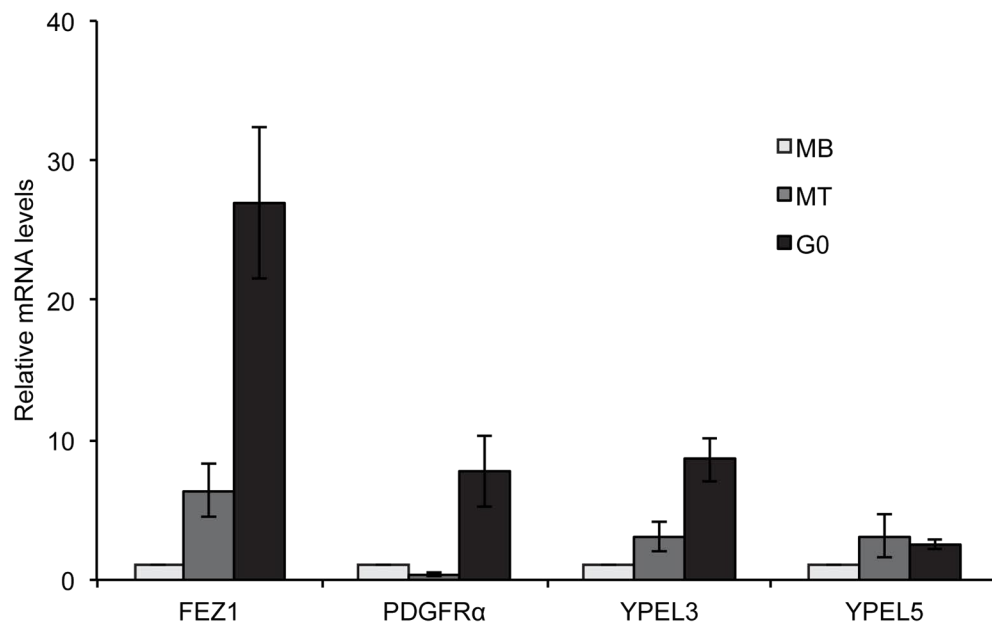

Supplement: Supplementary file 1 — Additional file 1: Figure S1. The transcriptome of G0 myoblasts displays a “cilium” signature. A. Venn Diagram showing overlap between genes that are differentially regulated as myoblasts enter quiescence (G0 altered) and “Ciliome”. Roughly 19% (p value: 1.717854e-19) of total genes altered in quiescence (G0) are linked to cilia by earlier studies. The significance of overlap was calculated using the formula “sum (dhyper (345:1896, 1747, 13253, 1896))”. B. qRT-PCR analysis to validate expression of genes linked to primary cilia found by microarray analysis. n = 3, Values represent mean ± s.e.m., p values are as follows: Fasciculation and elongation protein zeta-1 (Fez1)-0.000005, Platelet Derived Growth Factor Receptor Alpha (PDGFRα)-0.006, Yippee-like 3 (YPEL3)-0.001, Yippee-like 5 (YPEL5)-0.003. [file 12860_2020_266_MOESM1_ESM.pdf]

**Fig. S2**

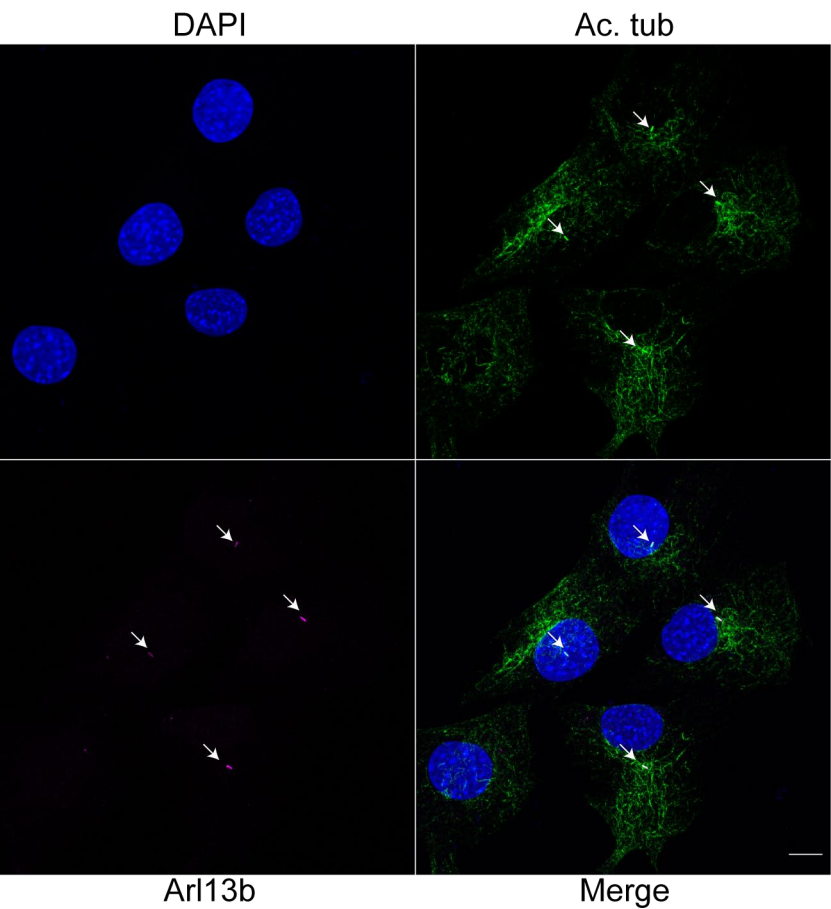

Supplement: Supplementary file 2 — Additional file 2: Figure S2. Visualization of primary cilia using immunofluorescence. Proliferating C2C12 myoblasts (MB) were fixed as described and immuno-stained using antibodies against Acetylated tubulin (Ac. Tub) (green), and Arl13b (Magenta). Acetylated tubulin showed robust staining and allowed detection of bona fide (Arl13b+) primary cilia and hence was used for further assays. [file 12860_2020_266_MOESM2_ESM.pdf]

DAPI

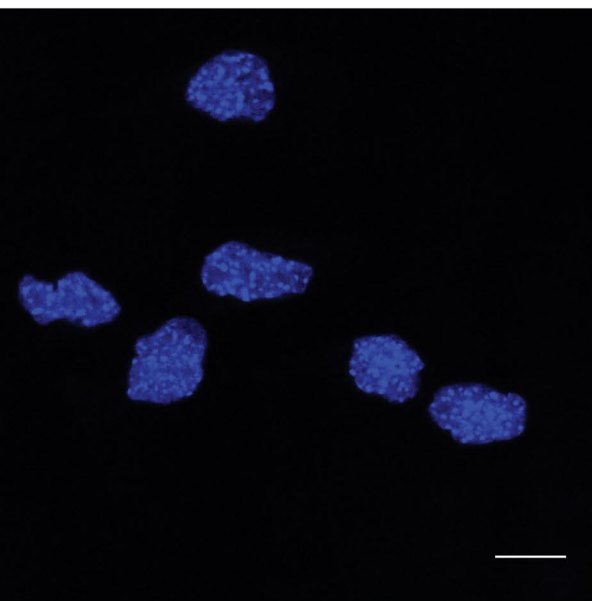

Myogenin

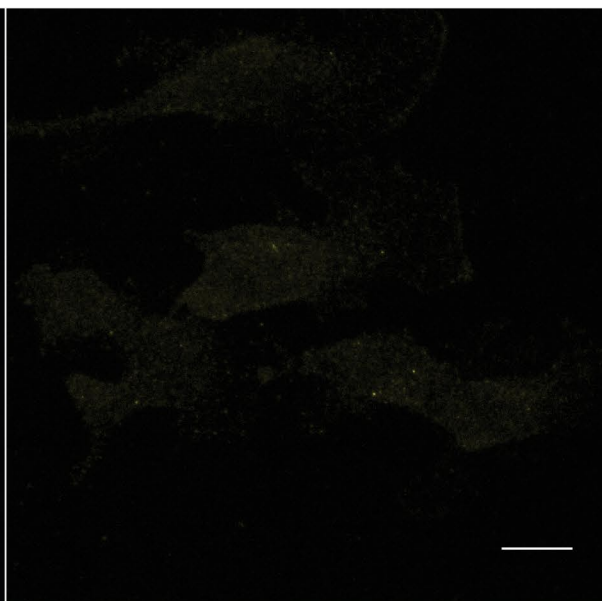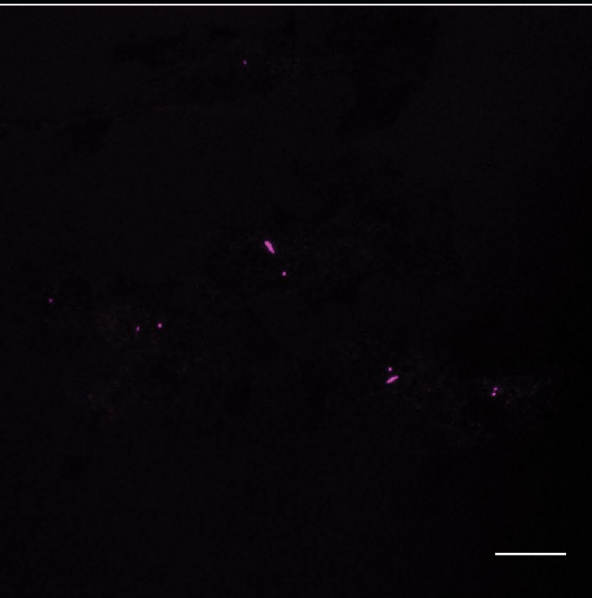

Ac. Tubulin

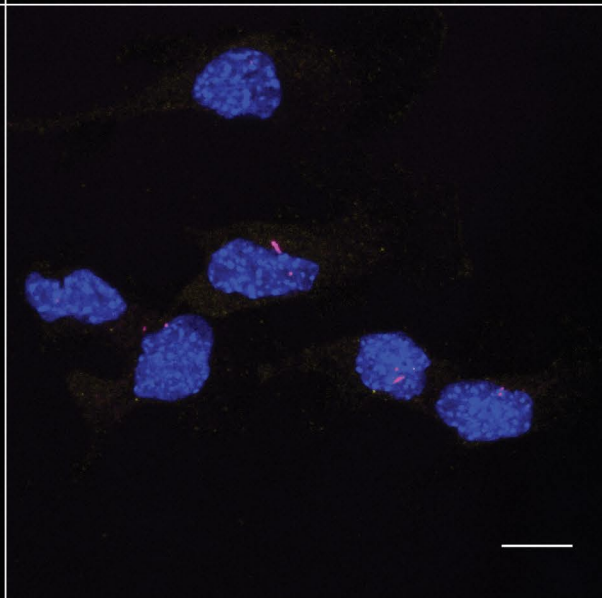

MERGE

Supplement: Supplementary file 4 — Additional file 4: Figure S4. Reserve cells isolated from differentiated cultures do not express Myogenin. 5-day differentiated C2C12 cultures were mildly trypsinised to remove myotubes, enriching the adherent undifferentiated mononuclear reserve cells. These cells were immunostained to confirm absence of Myogenin and primary cilia were detected using Acetylated tubulin (Ac.Tub). (Scale bar, 10 μm.) [file 12860_2020_266_MOESM4_ESM.pdf]

**Fig. S5**

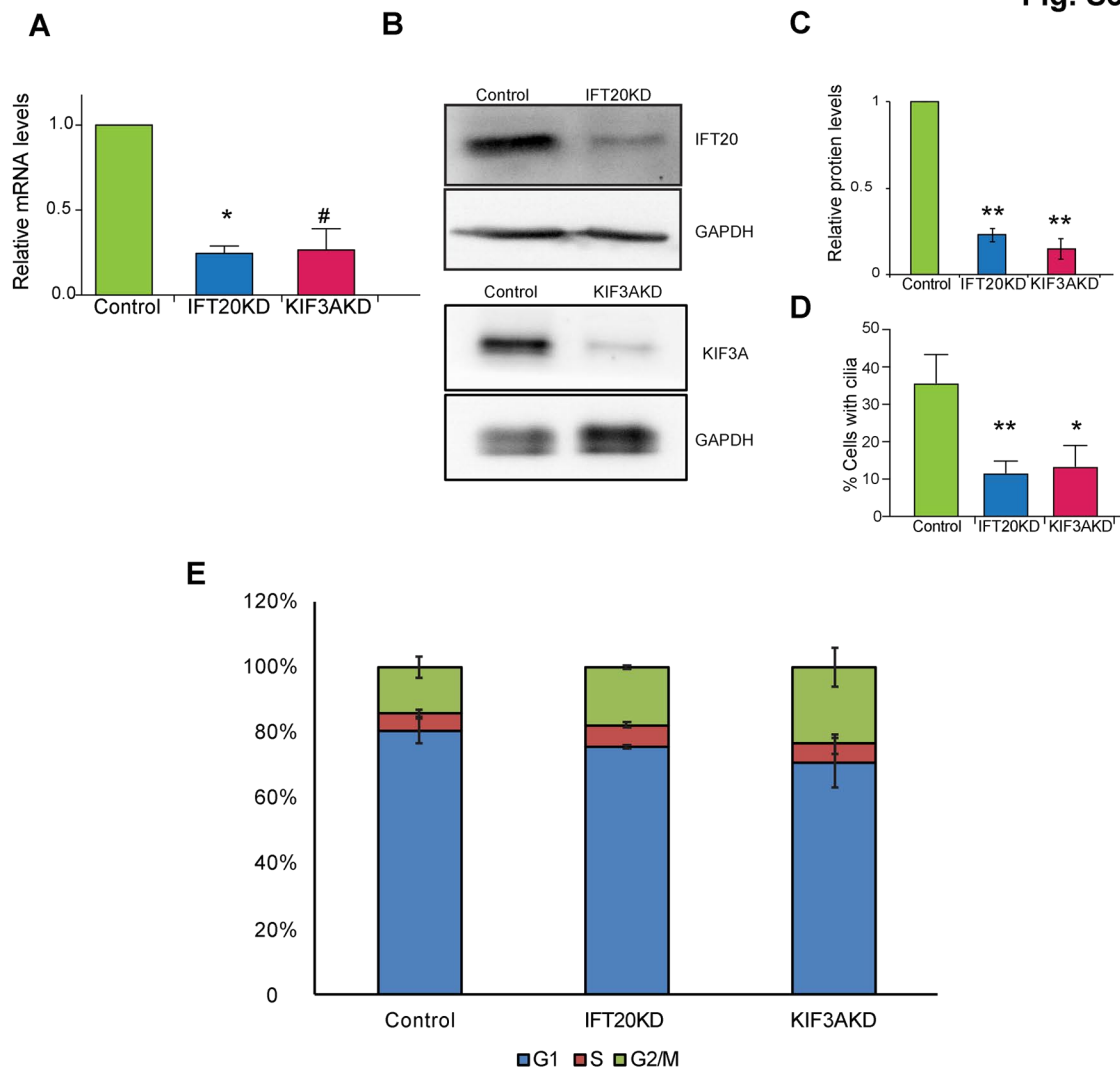

Supplement: Supplementary file 5 — Additional file 5: Figure S5. Key cell cycle effects in quiescent IFT88KD myoblasts were validated using RNAi against 2 other IFT targets. C2C12 myoblasts were transfected with siRNAs targeting IFT20 or KIF3A to block ciliogenesis, and were analyzed for effects of knockdown on proliferation and quiescence. Non-targeting siRNA was used as control. A. qRT-PCR demonstrates efficient knockdown of respective target mRNA levels 48 h after transfection. Values represent mean ± s.e.m., *p < 0.05, **p < 0.01, #p = 0.054, N = 3. B. Western blotting was used to demonstrate efficient knockdown of respective target protein levels. Values represent mean ± s.e.m., *p < 0.05, **p < 0.01, N = 3. C. Quantification of immunoblotting data shown in (B). Values represent mean ± s.e.m., **p < 0.01, N = 3. D. Primary cilia were visualized by immunofluorescence labelling of Acetylated tubulin (Ac. tubulin) in siRNA treated cells cultured in quiescence-inducing conditions. Knockdown of both IFT20 and KIF3A caused reduction in frequency of ciliated cells. Values represent mean ± s.e.m., *p < 0.05, **p < 0.01, N ≥ 3. E. Quantification of experiment described in (Fig. 2l) showing shifts in proportion of cells in different cell cycle stages (G1, S, G2/M). Values represent mean ± s.e.m., N = 3, p-values are as follows: IFT20KD – G1 = 0.29, S = 0.33, G2/M = 0.23; KIF3AKD – G1 = 0.07, S = 0.44, G2/M = 0.055. [file 12860_2020_266_MOESM5_ESM.pdf]

**Fig. S7**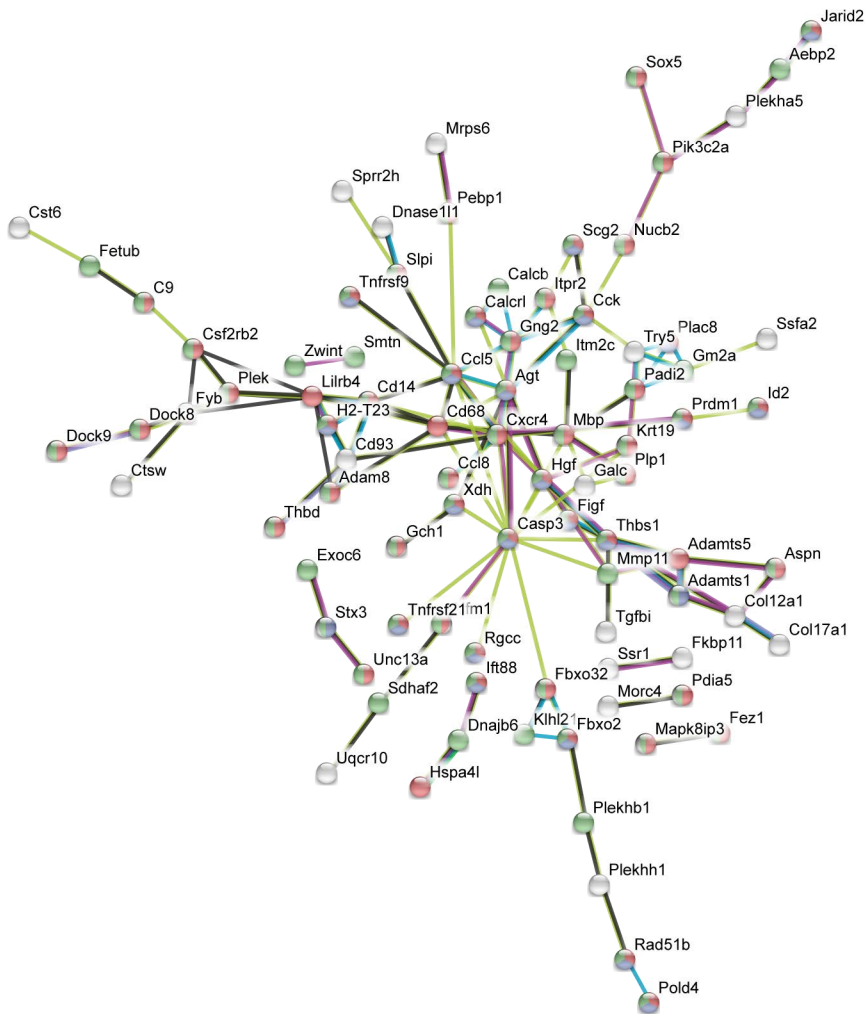

Supplement: Supplementary file 7 — Additional file 7: Figure S7. STRING network of genes that are down-regulated in IFT88KD myoblasts at conditions of G0 shows sparse network. In contrast to the network seen in up-regulated genes, the down-regulated genes in IFT88KD G0 myoblasts display a sparser interaction network. Nodes (genes) are coloured to depict the most significant Gene Ontology classes detected: Red- response to stimulus (GO:0050896, FDR: 5.72e-09); Blue - Regulation of cell population proliferation (GO:0042127, FDR: 1.22e-06); Green - Biological regulation (GO:0065007, FDR: 1.38e-06). The image does not display disconnected nodes. [file 12860_2020_266_MOESM7_ESM.pdf]

**A**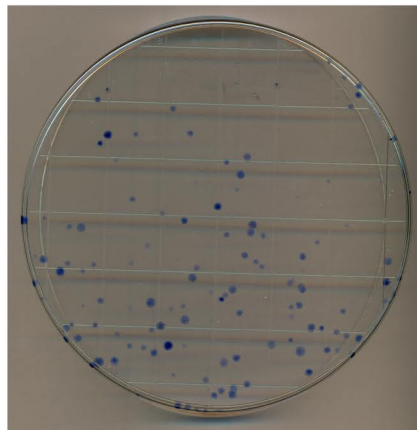

Control G0

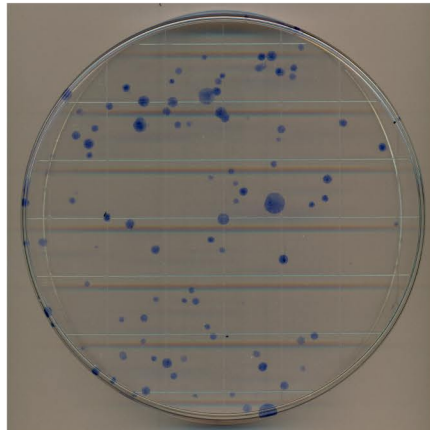

IFT88KD G0

**B**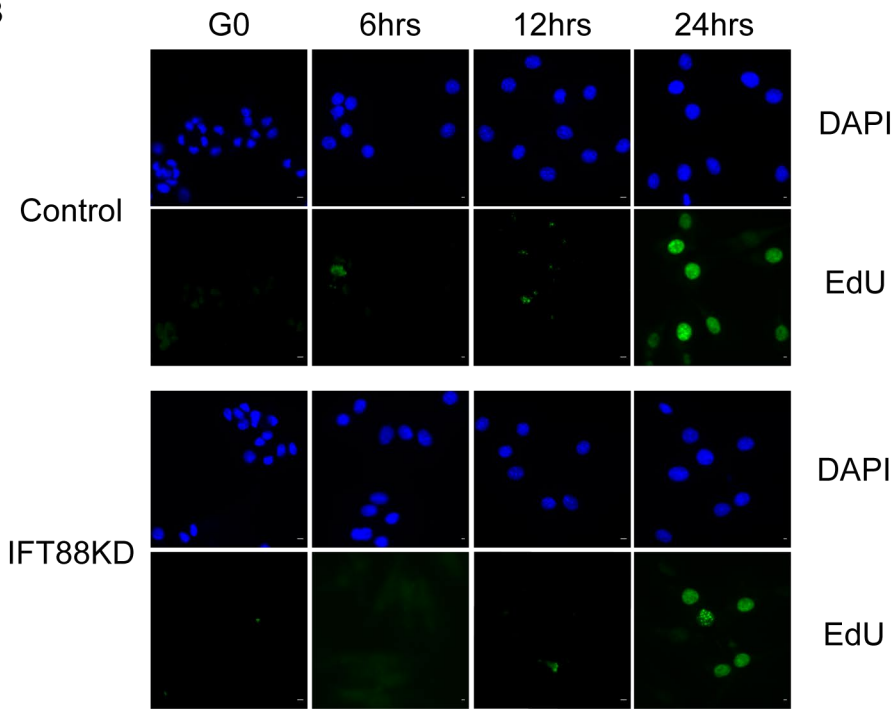

Supplement: Supplementary file 8 — Additional file 8: Figure S8. Representative images for Fig. 4a and b. A. Representative images for CFU Assay shown in Fig. 4a. IFT88KD and Control G0 myoblasts were harvested from suspension and replated at clonal density for analysis of colony forming potential (% CFU). IFT88KD myoblasts showed reduced self-renewal when compared to control cells. B. Representative images for EdU incorporation assay on reactivating myoblasts shown in Fig. 4b. IFT88KD and Control G0 myoblasts were harvested from suspension and replated for reactivation into cell cycle. Cells were pulsed with EdU and harvested at denoted time points for immunofluorescence analysis. IFT88KD myoblasts showed fewer cells in S phase (EdU+) than Control cells. [file 12860_2020_266_MOESM8_ESM.pdf]

**Fig. S9**

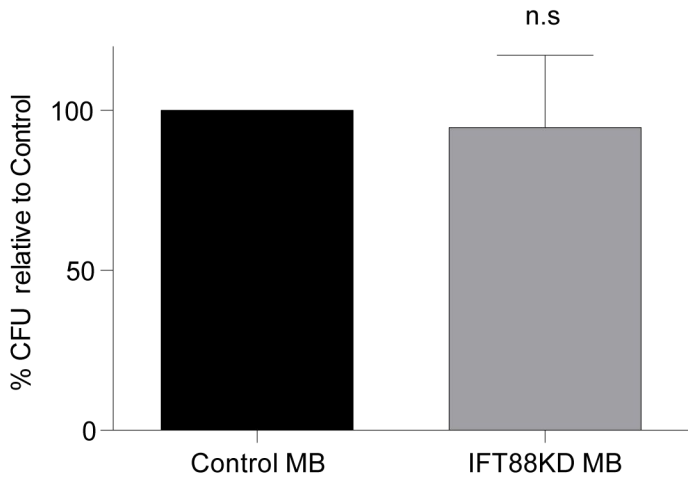

Supplement: Supplementary file 9 — Additional file 9: Figure S9. Self-renewal of proliferating myoblast cultures is not affected by IFT88 knock- down. Colony formation assay on IFT88KD myoblasts under proliferating conditions expressed as percentage Colony Forming Units relative to Control. [file 12860_2020_266_MOESM9_ESM.pdf]

**Fig. S10**

DAPI

Ac. tub

Control G0

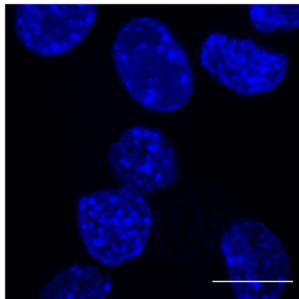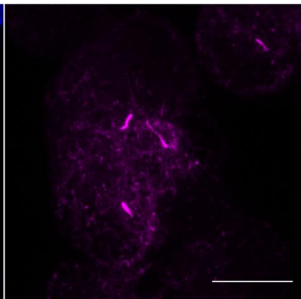

IFT88KD G0

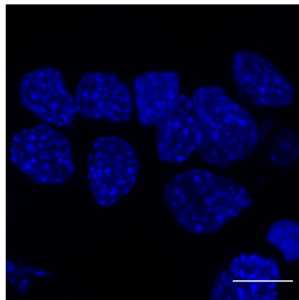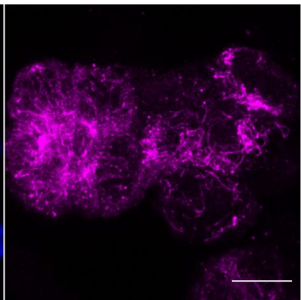

Supplement: Supplementary file 10 — Additional file 10: Figure S10. Knockdown efficiency for the p27 mVenus assay was validated using a parallelly transfected C2C12 culture. C2C12 cells that were transfected with either Control siRNA or siRNA targeting IFT88 were placed in suspension culture to induce G0. After 48 h, cells were harvested and fixed for immunofluorescence. Acetylated Tubulin (Ac.tub) marks primary cilia. No cilia were detected on IFT88KD cells examined, demonstrating efficient knockdown. [file 12860_2020_266_MOESM10_ESM.pdf]
