# Supplementary material for: The primary cilium dampens proliferative signaling and represses a G2/M transcriptional network in quiescent myoblasts
Source: BMC Mol Cell Biol. 2020 Apr 15;21:25. doi: 10.1186/s12860-020-00266-1 (PMC7161131; doi:10.1186/s12860-020-00266-1)
Supplement: Supplementary file 6 — Additional file 6: Figure S6. Heat map of differentially regulated genes in IFT88KD vs Control in 3 cell states. Rows are centered; unit variance scaling is applied to rows. Rows represent individual genes, and are clustered using correlation distance and average linkage. Columns represent different samples (Proliferating myoblasts (MB), quiescent myoblasts(G0), and myoblasts 24 h after reactivation into cell cycle from quiescence (R24)) and are clustered using Manhattan distance and average linkage. The dendogram shows the distance between the different samples. The color code indicated on the right shows the range of expression values. Similarly behaving genes have been clustered together separated by the gaps. All the replicates of each sample cluster together. G0 and R2 are most similar, while MB showed more variation and fewer altered genes [file 12860_2020_266_MOESM6_ESM.pdf]

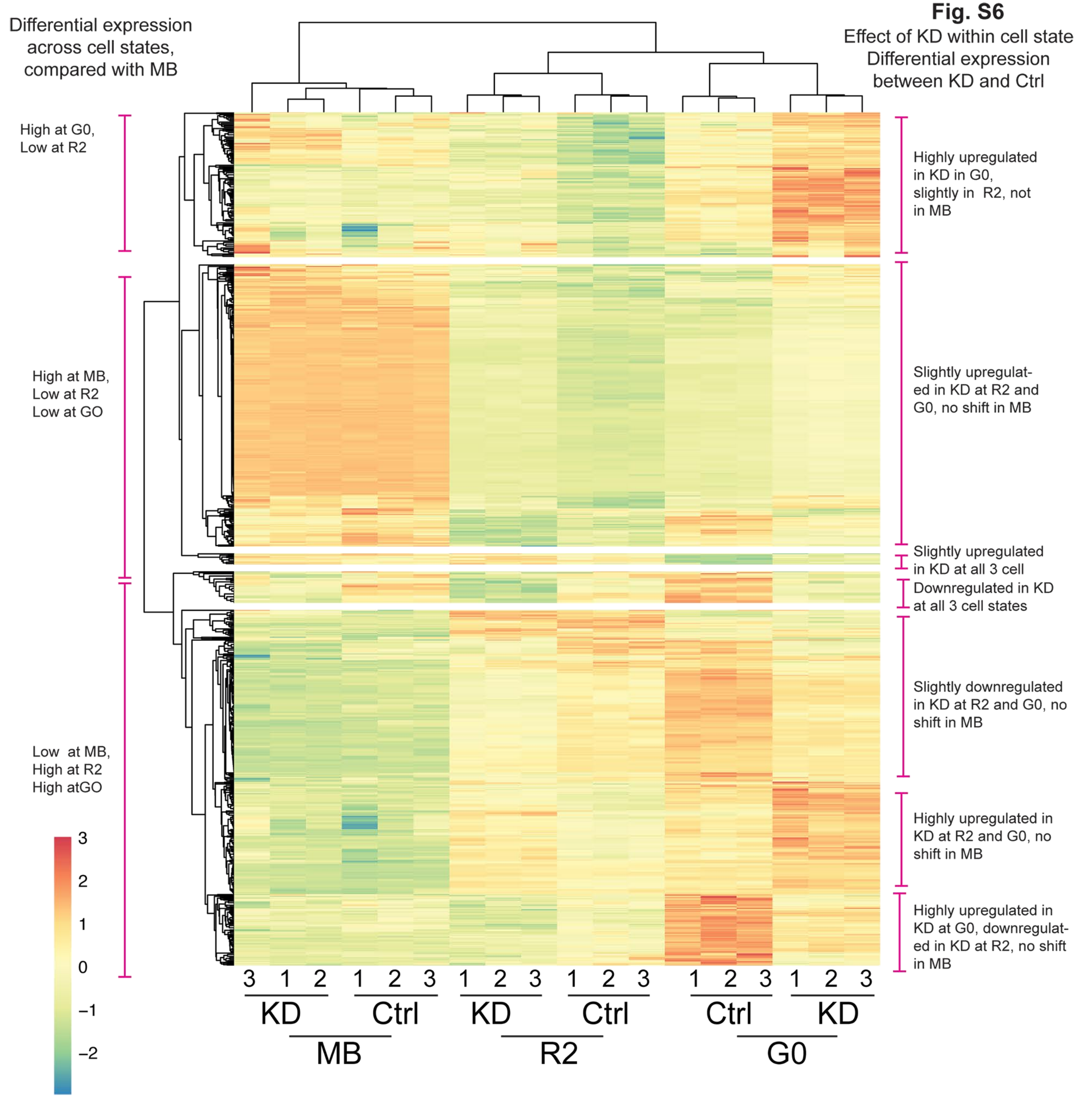
Fig. S6
Effect of KD within cell state
Differential expression
between KD and Ctrl
Differential expression
across cell states,
compared with MB
High at G0,
Low at R2
High at MB,
Low at R2
Low at GO
Low at MB,
High at R2
High atGO
Highly upregulated
in KD in G0,
slightly in R2, not
in MB
Slightly upregulat-
ed in KD at R2 and
G0, no shift in MB
Slightly upregulated
in KD at all 3 cell
Downregulated in KD
at all 3 cell states
Slightly downregulated
in KD at R2 and G0, no
shift in MB
Highly upregulated in
KD at R2 and G0, no
shift in MB
Highly upregulated in
KD at G0, downregulat-
ed in KD at R2, no shift
in MB
3
2
1
0
−1
−2
3 1 2 1 2 3 1 2 3 1 2 3 1 2 3 1 2 3
KD Ctrl KD Ctrl Ctrl KD
MB R2 G0
