# Supplementary material for: The primary cilium dampens proliferative signaling and represses a G2/M transcriptional network in quiescent myoblasts
Source: BMC Mol Cell Biol. 2020 Apr 15;21:25. doi: 10.1186/s12860-020-00266-1 (PMC7161131; doi:10.1186/s12860-020-00266-1)
Supplement: Supplementary file 3 — Additional file 3: Figure S3. Reduction of proliferation markers during quiescence entry by suspension culture. Representative Western Blot images showing reduction in levels of proliferation markers Ki67 and Cyclin A2 (CCNA2) during entry by 24 h into suspension culture to induce quiescence. In contrast, the transcript levels of the Growth Arrest Specific (GAS) gene PDGFRα, which has been reported to localize to the cilium, increase during this time period. [file 12860_2020_266_MOESM3_ESM.pdf]

Fig. S3

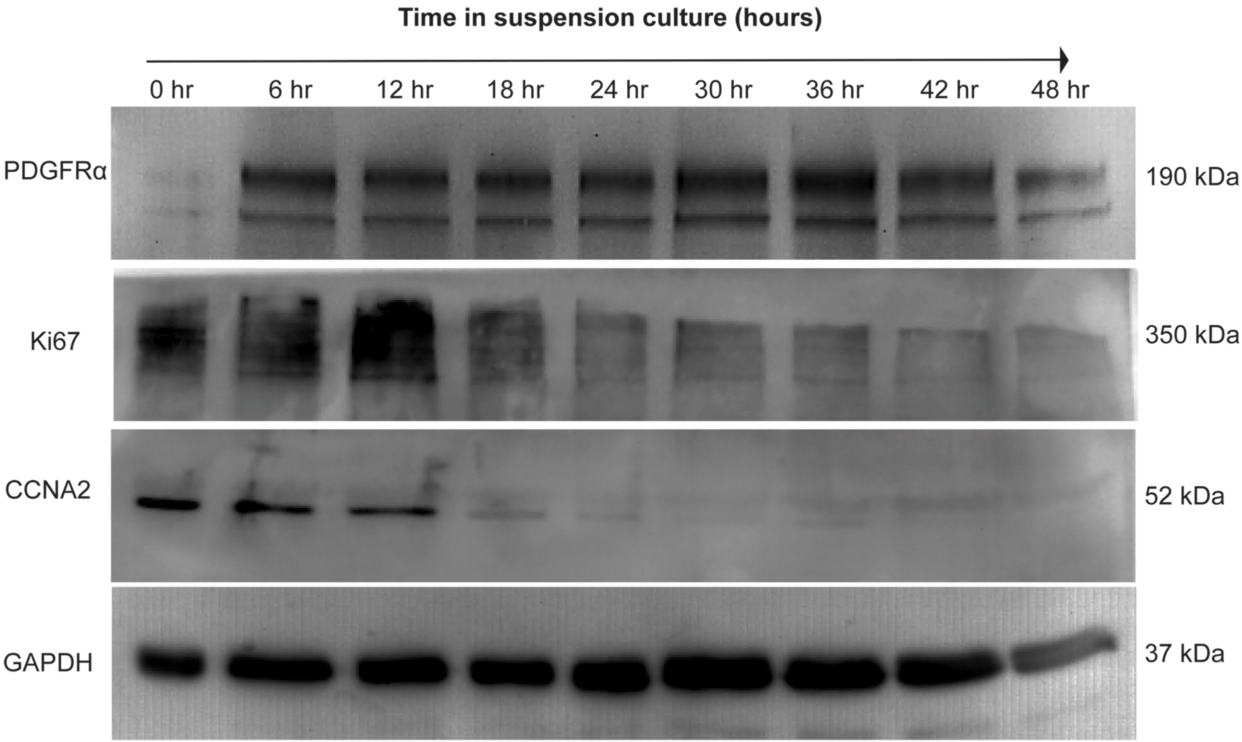
Time in suspension culture (hours)
0 hr
6 hr
12 hr
18 hr
24 hr
30 hr
36 hr
42 hr
48 hr
PDGFRα
190 kDa
Ki67
350 kDa
CCNA2
52 kDa
GAPDH
37 kDa
